# Preventive Effect of Boiogito on Metabolic Disorders in the TSOD Mouse, a Model of Spontaneous Obese Type II Diabetes Mellitus

**DOI:** 10.1093/ecam/nep012

**Published:** 2011-06-05

**Authors:** Tsutomu Shimada, Tomoko Akase, Mitsutaka Kosugi, Masaki Aburada

**Affiliations:** ^1^Research Institute of Pharmaceutical Sciences, Musashino University, 1-1-20, Shin-machi, Nishi-Tokyo-shi, Tokyo 202-8585, Japan; ^2^Graduate School of Medicine, The University of Tokyo, 7-3-1 Hongo, Bunkyo-ku, Tokyo 113-0033, Japan; ^3^Department of Clinical Pharmacy, Graduate School of Natural Science and Technology, and Department of Hospital Pharmacy, School of Medicine, Kanazawa University, 13-1 Takara-machi, Kanazawa 920-8641, Japan

## Abstract

“Boiogito” is a Kampo preparation which has been used since ancient times in patients with obesity of the “asthenic constitution” type, so-called “watery obesity”, and its effect has been recognized clinically. In this study, we investigated the anti-obesity effect of Boiogito in the TSOD (Tsumura Suzuki Obese Diabetes) mouse, a model of spontaneous obese type II diabetes mellitus. Boiogito showed a significant anti-obesity effect in TSOD mice by suppressing body weight gain in a dosage-dependent manner. In addition, Boiogito showed significant ameliorative effects on features of metabolic syndrome such as hyperinsulinemia, fasting hyperglycemia and abnormal lipid metabolism. Regarding lipid accumulation in TSOD mice, Boiogito showed a significant suppressive effect on accumulation of subcutaneous fat, but the effect on the visceral fat accumulation that constitutes the basis of metabolic syndrome was weak, and the suppressive effect on insulin resistance was also weak. Furthermore, Boiogito did not alleviate the abnormal glucose tolerance, the hypertension or the peripheral neuropathy characteristically developed in the TSOD mice. In contrast, in the TSNO (Tsumura Suzuki Non-Obesity) mice used as controls, Boiogito suppressed body weight gain and accumulation of subcutaneous and visceral fat. The above results suggested that Boiogito is effective as an anti-obesity drug against obesity of the “asthenic constitution” type in which subcutaneous fat accumulates, but cannot be expected to exert a preventive effect against various symptoms of metabolic syndrome that are based on visceral fat accumulation.

## 1. Introduction

Visceral fat accumulation is considered to induce insulin resistance and has been proposed as the cause of the so-called “metabolic syndrome" (MS), a major risk factor for the ischemic cardiac and cerebrovascular diseases that rank high as causes of death [1, 2]. At present, it is said that in the Japanese population aged 40 years old or more, one of two males and one of five females are MS patients or would-be MS patients; thus the importance of MS prevention has been re-realized [3].

In Kampo medicine, which originated in China in ancient times and was developed uniquely in Japan, the so-called “Obesity" is also treated; preparations prescribed include Daisaikoto, Saikokaryukotuboreito, Tokakujokito, Daiobotanpito, Bofutsushosan, Tudosan, Kumibinroto, Boiogito, and so forth, [4]. In general, obesity falls into two broad categories depending on its cause. In Western medicine, obesity is classified into two types characterized by either visceral or subcutaneous fat accumulation. On the other hand, in Kampo medicine, obesity is classified into the “robust constitution"-type and the “asthenic constitution"-type. In Western medicine, the obesity causing MS is of the visceral fat accumulation type, corresponding to obesity of the “robust constitution" type (otherwise known as “firm obesity") in Kampo medicine. Of the above Kampo prescriptions, Daisaikoto and Bofutsushosan are used for the high physical energy type obesity, that is, obesity of the “robust constitution" type, and we have already reported that Daisaikoto and Bofutsushosan have an effect on “visceral fat accumulation type" obesity and show a preventive effect on various metabolic disorders such as abnormal lipid metabolism, hyperinsulinemia, insulin-resistant hypertension and peripheral neuropathy [5, 6]. In this study we investigated the preventive effect of Boiogito, which has been prescribed for obesity of the “asthenic constitution" type, on obesity and various metabolic diseases in the TSOD (Tsumura Suzuki Obese Diabetes) mouse [7–11], a model of spontaneous obese type II diabetes mellitus. TSOD mice were established as a multi-gene obese type-II diabetes model by selecting individual animals that developed obesity and diabetes from among ddY mice and repeating brother-sister inbreeding using the body weight and male urinary glucose level as indices.

## 2. Subjects and Methods

### 2.1. Experimental Animals

Male TSOD mice were used as a model of spontaneous obese type II diabetes mellitus, and male TSNO (Tsumura Suzuki Non Obesity) mice were used as controls. The TSOD and TSNO mice aged 3 weeks (20 animals each) were purchased from the Institute for Animal Reproduction (Ibaragi Prefecture). The animals were kept in a dedicated animal room under controlled conditions (temperature: 23 ± 2°C, humidity: 55 ± 10%, light on for 12 h and off for 12 h per day). After 1 week of acclimation, the experiment was started. During the acclimation period, the animals were given Powder Feed MF (Oriental Yeast Co., Ltd) and water *ad libitum*, but the normal feed was switched to the test feed from the age of 4 weeks. The test feed, prepared by mixing the normal feed with Boiogito, was given for 8 weeks. All the animal experiments were performed in compliance with the rules for animal experimentation established at Musashino University.

### 2.2. Test Drug

**Figure 1:**
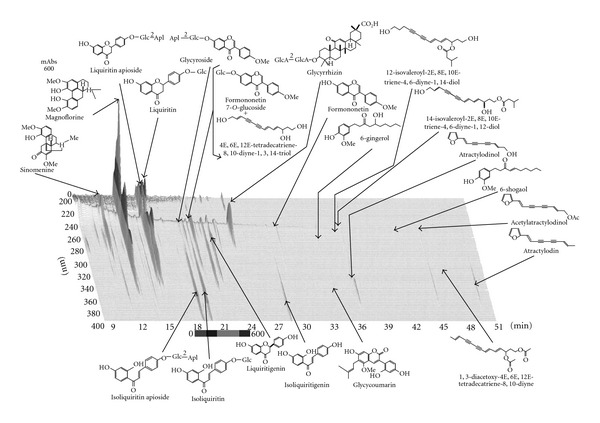
Analysis by three-dimensional HPLC of major chemical compounds included in Boiogito extracts.

The Boiogito extract powder was supplied by Tsumura & Co. (Tokyo). This herbal prescription in Table 1 was added to water of 10 times of its quantity and extracted at 100°C for 1 h. The extract was filtered and spray-dried to obtain the dry extract powder.  Figure 1 shows the 3D HPLC chart of the Boiogito extract powder. The test feed was prepared by mixing the normal feed, Powder Feed MF, with the Boiogito extract powder thoroughly so that the uniform concentration of Boiogito was 1.0% or 3.0%.

### 2.3. Mode of Administration

After 1 week of acclimation, the TSOD and TSNO mice aged 4 weeks were weighed and grouped so that the body weight distribution was not markedly different among the groups. The control groups were given Powder Feed MF *ad libitum*, and the drug treatment groups were given the feed containing 1.0% Boiogito (low-dosage group) or the feed containing 3.0% Boiogito (high-dosage group) *ad libitum*.

### 2.4. Body Weight Gain and Feed Intake

The body weight was measured once a week. The feed intake was measured from 1 week after starting the experiment and calculated as the mean daily value in each cage by deducting the remaining feed amount and the spilled feed amount from the feed amount filled into the feed container on the day before and dividing the difference by the number of animals in the cage.

### 2.5. Determination of Amounts of Visceral and Subcutaneous Fat

At 8 weeks after starting administration of the test feed (at the age of 12 weeks), each mouse was anesthetized with Nembutal (50 mg/kg, i.p.) and placed within computerized tomography (X-ray CT) equipment used for experimental animals (La Theta, Aloka Co., Ltd, Tokyo). The amounts of visceral and subcutaneous fat were determined for the whole body and at individual sites by scanning at intervals of 1.5 mm.

### 2.6. Blood Biochemical Tests

At 8 weeks after starting administration of the test feed (at the age of 12 weeks), each mouse was subjected to blood sampling from the orbital cavity venous plexus under non-fasting conditions without anesthesia. This blood was centrifuged to obtain plasma; blood glucose, total cholesterol (TC) and triacylglycerol (TG) levels were determined using biochemical test kits (Wako Pure Chemical Industries, Ltd. Osaka) and the insulin level was assayed using Rebis Insulin-Mouse-T (ELISA Insulin Kit, Shibayagi Co., Ltd., Tokyo).

### 2.7. Glucose Tolerance Test

At 8 weeks after starting administration of the test feed (at the age of 12 weeks), each mouse was made to fast for at least 18 h and then glucose was administered orally (2 g/kg). Blood samples were taken immediately before administration of glucose and at 30, 60, 120 and 180 min after administration of glucose from the orbital cavity venous plexus. The blood taken was centrifuged to obtain the plasma, followed by determination of plasma glucose levels.

### 2.8. Blood Pressure

At 8 weeks after starting administration of the test feed (at the age of 12 weeks), each mouse was restrained in a restraint device (THC-2, Softron Co., Ltd, Tokyo), and the body temperature was maintained at 37°C. The tail was inserted (up to the base) into the tail cuff of a non-invasive blood pressure meter (BP-98A, Softron Co., Ltd.) to determine the systolic blood pressure, diastolic blood pressure and mean blood pressure.

### 2.9. Pain Test

The peripheral neuropathy was determined using the foot pinch method reported by Suzuki et al. [12]. At 8 weeks after starting administration of the test feed (at the age of 12 weeks), the proximal tarsal part of the hind limb metatarsal region was clipped with an artery clamp (BHO20R, pressure: 300 g, Bulldog Clamp, Johns Hopkins, Tokyo), and the time until the mouse bit the clamp on the hind limb was measured as the latent time to response. The average of the latent time values of the two hind limbs was considered to be the latent time of the mouse concerned.

### 2.10. Statistical Processing

In each experiment, the inter-group difference was tested for significance by Dunnett's multiple comparison with a significance level of 5%.

## 3. Results

### 3.1. Body Weight Gain and Feed Intake

**Figure 2:**
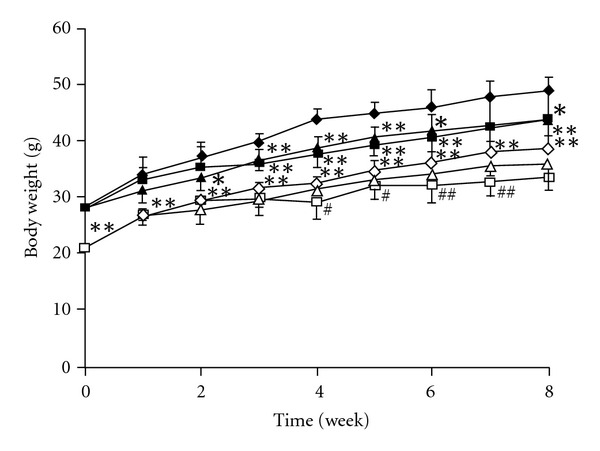
The effect of Boiogito on the body weight in mice. Filled diamond: TSOD control, filled square: TSOD Boiogito 1%, filled triangle: TSOD Boiogito 3%, open diamond: TSNO control, open square: TSNO Boiogito 1%, open triangle: TSNO Boiogito 3%. Data represent the mean ± SD of five to seven animals. *P < .05 and **P < .01 significantly differs from the TSOD control group. ^#^ *P* < .05, ^##^ *P* < .01, significantly differs from the TSNO control group.

Figure 2 shows the body weight gains in the TSOD and TSNO mouse groups. The body weight gain was higher in the TSOD mouse control group than in the TSNO mouse control group from the start of the experiment, and the TSOD mice were obese. In the TSOD mouse groups, the body weight gain was significantly suppressed in the Boiogito low-dosage group from 4 weeks after starting the experiment and in the Boiogito high-dosage group from immediately after starting the experiment as compared with the control group. In the TSNO mouse group, the body weight gain was significantly suppressed in both the low- and high-dosage groups from around 4 weeks after starting the experiment.

The feed intake was higher in the TSOD mouse groups than in the TSNO mouse group throughout the experiment period but no significant difference was seen between any two TSOD mouse groups and between any two TSNO mouse groups (feed intake at the age of 6 weeks: 3.5 ± 0.2 g/day/body in the TSNO control group, 3.4 ± 0.6 g/day/body in the TSNO Boiogito low-dosage group and 3.3 ± 0.3 g/day/body in the TSNO Boiogito high-dosage group; 4.3 ± 0.1 g/day/body in TSOD control group, 3.8 ± 0.6 g/day/body in the TSOD Boiogito low-dosage group and 4.3 ± 0.4 g/day/body in the TSOD Boiogito high-dosage group)

### 3.2. Changes in Amounts of Visceral and Subcutaneous Fat

**Figure 3:**
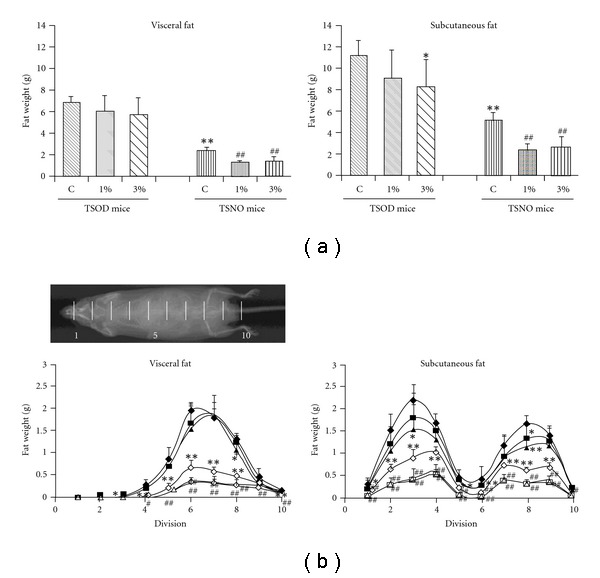
Effects of Boiogito on fat accumulation in TSOD and TSNO mice. (a) Total amounts of visceral and subcutaneous fat at 8 weeks after starting the experiment. (b) By-site accumulation of visceral and subcutaneous fat at 8 weeks after starting the experiment Filled diamond: TSOD control, filled square:TSOD Boiogito 1%, filled triangle: TSOD Boiogito 3%, open diamond: TSNO control, open square: TSNO Boiogito 1%, open triangle: TSNO Boiogito 3%. Data represent the mean ± SD of five to seven animals. *P < .05, **P < .01, significant differences from the TSOD control group. ^#^ *P* < .05, ^##^ *P* < .01, significant differences from the TSNO control group.

Figure 3(a) shows the total visceral and subcutaneous fat at 8 weeks after starting the experiment, and Figure 3(b) shows the by-site accumulation status of visceral fat and subcutaneous fat. As clearly seen in Figure 3(a), total visceral fat and total subcutaneous fat accumulated significantly more in the TSOD mouse control group than in the TSNO mouse control group. In the TSOD mouse groups, the Boiogito treatment did not influence the total amount of visceral fat but significantly suppressed accumulation of subcutaneous fat. As shown in Figure 3(b), which shows the by-site accumulation of subcutaneous fat 8 weeks after starting the experiment, Boiogito caused dose-dependent suppression of accumulation of subcutaneous fat around the scapula and hip joint, and a significant difference was seen in the Boiogito high-dosage group. Conversely, in the TSNO mouse groups, a significant suppressive effect was seen on accumulation of total visceral fat, total subcutaneous fat, by-site visceral fat and by-site subcutaneous fat in both the Boiogito low- and high-dosage groups.

### 3.3. Blood Biochemical Tests

Table 2 shows blood biochemical test values in the TSOD mouse groups and TSNO mouse groups at the time of completing the experiment.

The fasting blood glucose level, TC level and insulin level were higher in the TSOD mouse control group than in the TSNO mouse control group. In the TSOD mouse groups, the TC and TG levels were suppressed in the Boiogito treatment groups in a dosage-dependent manner, and the difference from the control group was significant for TC level in the high-dosage group and for TG level in both the low- and high-dosage groups. There were no changes in blood glucose level after feed intake related to Boiogito treatment, but the fasting blood glucose level was significantly lower in both the Boiogito low- and high-dosage groups than in the control group. The insulin level was lower only in the Boiogito low-dosage group. Conversely, in the TSNO mouse group no parameter changed in relation to Boiogito treatment.

### 3.4. Glucose Tolerance Test

At 8 weeks after starting administration of the test feed, each mouse was made to fast for one night and then given glucose orally (2 g/kg). From the time-course changes in blood glucose level up to 180 min after glucose administration, the “Glucose area under the concentration curve" (AUC) was calculated.  Table 3 shows the results. The AUC was significantly higher in the TSOD mouse control group than in the TSNO mouse control group. There was no marked difference from the control group in either the TSOD mice or TSNO mice treated with Boiogito.

### 3.5. Blood Pressure

Table 4 shows the systolic blood pressure, diastolic blood pressure and mean blood pressure in each group of the TSOD and TSNO mice.

The systolic blood pressure, diastolic blood pressure and mean blood pressure were significantly higher in the TSOD mouse control group than in the TSNO mouse control group. In both the TSOD mice and TSNO mice, the Boiogito treatment had no substantial influence on systolic blood pressure, diastolic blood pressure or mean blood pressure.

### 3.6. Pain Test

**Figure 4:**
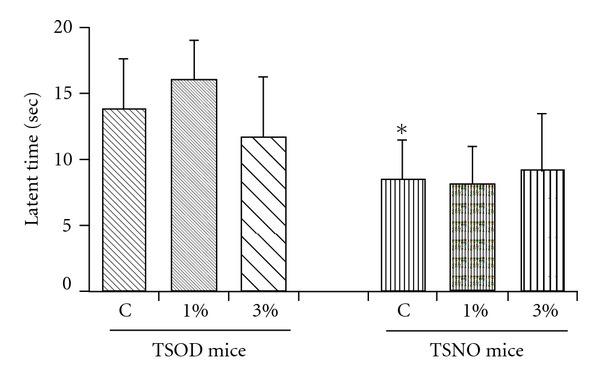
The effect of Boiogito on the peripheral neuropathy. Data represent the mean ± SD of five to seven animals. *P < .05, significantly differs from the TSOD control group.

Figure 4 shows the pain test results in each group of TSOD mice and TSNO mice.

The latent time to response was significantly longer in the TSOD mouse control group than in the TSNO mouse control group. It was confirmed that the TSOD mouse control group developed neuropathy. In both the TSOD mice and TSNO mice, the Boiogito treatment had no substantial influence on latent time to response.

## 4. Discussion

In recent years, the number of obese people has increased rapidly due to Westernization of eating habits and development of means of transportation, but on the other hand, excessive dieting for beauty/shape maintenance and various healthy foods have become popular among females. In general, obesity is classified into the visceral fat accumulation type (male type) and the subcutaneous fat accumulation type (female type) [13], and subsequent disease progression and therapy are different depending on the type. MS highlighted in the world has been reported to be caused especially by visceral fat accumulation.

In Kampo medicine, obese patients are divided into the “robust constitution" type and the “asthenic constitution" type, and various Kampo preparations tailored to each type are prescribed. Many obese patients of the “robust constitution" type have obesity of the so-called “visceral fat accumulation type" and are generally big-boned, tend to be constipated, and have a plump lower abdomen. Conversely, many obese patients of the “asthenic constitution" type have obesity of the so-called “subcutaneous fat accumulation type" and tend to be fair-skinned, soft-muscled, edematous/obese, and sweat easily [14].

As a part of programs to develop preventive or therapeutic drugs against various symptoms of the MS, we have investigated the effects of Kampo preparations, which have been used against obesity from ancient times, using multi-factor genetic TSOD mice that show obesity characterized by spontaneous accumulation of abdominal and subcutaneous fat, and develop various metabolic disorders such as abnormal glucose/lipid metabolism and hypertension. As mentioned above, we have already clarified that Daisaikoto and Bofutsushosan, which are used against obesity of the “robust constitution" type, show preventive effects against various symptoms of MS, although effects vary [5, 6]. MS is conceived of as a disease state characterized by excessive energy due to overeating and shortage of exercise and corresponds to the “robust constitution" type in Kampo medicine, so the above effects seen with Daisaikoto and Bofutsushosan make theoretical sense. On the other hand, Boiogito used in this study is the Kampo preparation described in the Chinese ancient literature as “Jin Kui yao lue", which contains six crude drugs and is used for treatment of obesity of the “asthenic constitution" type, that is, patients who are fair-skinned, soft-muscled, edematous (rich in subcutaneous fat) and easily susceptible to edema [4, 15]. Boiogito was reported to lower blood glucose in a study using mice with streptozotocin-induced diabetes [16, 17]. Furthermore, in ovariectomized rats, Boiogito showed an anti-obesity effect, and partial involvement of TNF*α* was suggested as the mechanism of action [18].

In this study, to investigate the preventive effect of Boiogito on obesity and various metabolic diseases, we used TSOD mice aged 4 weeks in which obesity and various metabolic disorders had not yet developed, and TSNO mice were used as controls. When body weight gain in the TSOD mice and TSNO mice was compared, the TSOD mice showed significantly higher body weight gain from the start of the experiment, as reported previously, and apparently develop obesity. Boiogito suppressed body weight gain from the early stages of treatment in both the TSOD mouse groups and TSNO mouse groups. TSNO mice are normal mice developing no disease, and the suppressive effect of Boiogito on body weight gain in the TSNO mice (a 10% decrease in the high-dosage group as compared with the control group) was comparable with that in the TSOD mice (a 7% decrease in the high-dosage group as compared with the control group), suggesting that Boiogito shows an anti-obesity effect or suppressive effect on body weight gain not only in the obese state but also in the non-diseased (healthy) state. Since the main cause of obesity is fat accumulation, visceral and subcutaneous fat accumulations were examined using X-ray CT, by site. We found that the total visceral and subcutaneous fat accumulation were significantly higher in the TSOD mouse control group than in the TSNO mouse control group. The suppressive effects of Boiogito on this accumulation were investigated. In the TSOD mouse groups, the suppressive effect was significant for total subcutaneous fat accumulation in the high-dosage group, and in the TSNO mouse groups, the suppressive effect was significant for both total visceral fat accumulation and total subcutaneous fat accumulation in both the low- and high dosage groups. Furthermore, looking at by-site fat accumulation, in the TSOD mouse groups, Boiogito significantly suppressed subcutaneous fat accumulation around the scapula and hip joint in a dosage-dependent manner, and in the TSNO mouse groups, Boiogito significantly suppressed both visceral fat accumulation and subcutaneous fat accumulation throughout most of the body. These results suggest that the anti-obesity effect or suppressive effect of Boiogito on body weight gain is mainly due to the suppression of subcutaneous fat accumulation around the scapula and hip joint. Morishima et al. confirmed a decrease in subcutaneous fat in subjects given Boiogito [19] coinciding with the effect of Boiogito on subcutaneous fat accumulation seen in this study.

We next investigated the effects of Boiogito on the phenomena occurring after visceral fat accumulation such as insulin resistance, abnormal glucose tolerance, abnormal lipid metabolism, hypertension, and so forth. The fasting blood glucose level and insulin level were significantly higher in TSOD mice than in TSNO mice, on comparison of all corresponding groups. In TSOD mice, Boiogito treatment significantly suppressed the increase in fasting blood glucose levels in both the Boiogito low- and high dosage groups but significantly suppressed the increase in insulin levels only in the low-dosage group. No dosage-dependency was seen in this suppression, and the Boiogito treatment did not improve the abnormal glucose tolerance seen in the glucose tolerance test of the TSOD mice. Furthermore, the Boiogito treatment did not change the blood glucose level after feed intake. These results suggest that the effects of Boiogito on lowering blood glucose and improving insulin resistance are not potent. On the other hand, in the TSNO mice, the Boiogito treatment did not influence fasting blood glucose levels or insulin levels. As regards lipid metabolism, the TC level and TG level were significantly higher in the TSOD mice than in the TSNO mice on comparison of all corresponding groups. Regarding the abnormal lipid metabolism seen in TSOD mice, the Boiogito treatment significantly decreased the TC level in the high-dosage group and significantly decreased the TG level in the low- and high-dosage groups. Of the crude drugs contained in Boiogito, it is known that a water extract of *Zingiber officinale* shows an inhibitory effect on pancreatic lipases and an anti-obesity effect [20]; the ethanol extract lowers TG, lipoprotein and phospholipid levels in hyperlipidemic animals [21] and other components inhibit cholesterol synthesis in the liver [22] In addition, glycyrrhizin, a component of *Glycyrrhiza uralensis*, is known to improve hyperglycemia, hyperinsulinemia and abnormal glucose tolerance, and so forth, with high-dose administration in KK-A^y^ mice [23]. Furthermore, *Astragalus mongholicus* is known to improve blood glucose profile [17, 18]. It is thus apparent that *Z. officinal*, *G. uralensis* and *A. mongholicus* contribute greatly to the beneficial effects of Boiogito on abnormal glucose metabolism and abnormal lipid metabolism. Since, as mentioned, the effect of Boiogito on insulin resistance was not potent, we speculate that direct inhibition of lipid absorption might contribute to the effect of Boiogito on abnormal lipid metabolism.

In addition, Boiogito did not show any particular ameliorative effects on the hypertension and peripheral neuropathy that occur in TSOD mice. We speculate that, as noted, Boiogito did not show a definite beneficial effect on insulin resistance, this accounts for the absence of an effect on consequent symptoms such as hypertension and peripheral neuropathy.

In the TSNO mouse used as non-diseased model, Boiogito suppressed body weight gain, visceral fat accumulation and subcutaneous fat, but didn't influence the chemical parameters, abnormal glucose tolerance, blood pressure and pain test. As indicated above, *Z. officinale*, one component of Boiogito, shows an inhibitory effect on pancreatic lipases. Therefore, inhibition of lipid absorption by *Z. officinale* might be a factor in suppression effect of body weight gain.

As mentioned above, Boiogito showed an effect against obesity, but this effect was mainly attributable to a suppressive effect on subcutaneous fat accumulation. We suggest that a specific effect on “visceral fat accumulation type" obesity cannot be expected and that Boiogito should be used in females of the “asthenic constitution" type or patients with “watery obesity" according to the theory of Kampo medicine.

## Figures and Tables

**Table 1:** The ingredients of Boiogito formula

Crude drugs	Weight ratio (g)
*Astragalus mongkohcus* Bunge	5.0
*Sinomenium acutum* Rehder et Wilson	5.0
*Atractylodes lancea* De Candolle	3.0
*Zizyphusjujube* Miller var. *inermis* Rehder	3.0
*Glycyrrkiza uralensis* Fisher	1.5
*Zibgiber ojficinale* Roscoe	1.0

**Table 2:** Effects of Boiogito on biochemical parameters of plasma

	TSOD mice			TSNO mice		
	Control	1%	3%	Control	1%	3%
Feed-Glucose (mg/dl)	184 ± 41	194 ± 38	159 ± 20	188 ± 33	155 ± 15	164 ± 20
Fast-Glucose (mg/dl)	174 ± 18	121 ± 11**	139 ± 43*	87 ± 12**	95 ± 9	97 ± 12
TC (mg/dl)	228 ± 448	209 ± 43	172 ± 12*	140 ± 18**	154 ± 12	146 ± 6
TG (mg/dl)	234 ± 28	173 ± 20**	166 ± 35**	129 ± 20**	148 ± 33	151 ± 35
Insulin (ng/ml)	15.8 ± 15.3	2.4 ± 1.6*	9.0 ± 7.3	1.5 ± 1.3*	2.5 ± 1.9	1.9 ± 0.9

Data represent the mean ± SD of 5–7 animals.

*P < .05, **P < .01, significantly differences from the TSOD control group.

**Table 3:** Effect of Boiogito on AUC in the oral glucose tolerance test

AUC (mg/ml/min)	Control	Boiogito (1%)	Boiogito (3%)
TSOD mice	89.2 ± 16.4	76.2 ± 16.8	75.8 ± 20.1
TSNO mice	61.5 ± 8.4*	58.3 ± 3.2	55.1 ± 8.3

Data represent the mean ± SD of 5–7 animals.

*P < .05, Significantly different from the TSOD control group.

**Table 4:** The effect of Boiogito on blood pressure in mice

BP (mmHg)	TSOD mice			TSNO mice		
	Control	1%	3%	Control	1%	3%
Systolic BP	113 ± 12	102 ± 6	111 ± 13	99 ± 5*	95 ± 4	102 ± 9
Diastolic BP	78 ± 9	73 ± 5	74 ± 6	69 ± 4*	70 ± 5	72 ± 9
Mean BP	90 ± 9	83 ± 5	86 ± 5	79 ± 3*	78 ± 5	81 ± 9

Data represent the mean ± SD of 5–7 animals.

*P < .05, Significantly different from the TSOD control group.

BP: Blood pressure.
